# Assessment of lipid parameters and its association with risk of stage 1 hypertension: a cohort study in middle-aged and elderly Chinese

**DOI:** 10.18632/aging.206280

**Published:** 2025-07-14

**Authors:** Jiabin Lin, Junyan Yu, Chulin Huang, Diaozhu Lin, Feng Li, Yiqin Qi, Ying Liang, Chuan Wang, Leiqin Cai, Yongfeng Huang, Wanting Feng, Na Li, Guojuan Lao, Huisheng Xiao, Chuan Yang, Li Yan, Meng Ren, Kan Sun

**Affiliations:** 1 Department of Endocrinology, Sun Yat-sen Memorial Hospital, Sun Yat-sen University, Guangzhou 510120, People's Republic of China; 2 Baiyun Branch of Nanfang Hospital, Southern Medical University, Guangzhou 510599, People's Republic of China

**Keywords:** stage 1 hypertension, dyslipidemia, TG to HDL-C ratio, middle-aged and elderly Chinese, cohort study

## Abstract

Background: There is a correlation between dyslipidemia and elevated blood pressure, but the relationship between different lipid parameters and elevated blood pressure is inconsistent. Therefore, understanding the impact of dyslipidemia on blood pressure is significant for the prevention and control of ASCVD. Our study is aimed at assessing longitudinally the correlation between lipid parameters and the incidence of stage 1 hypertension in a large Chinese community population.

Methods: This community-based cohort study conducted from 2011 to 2014–2016 included 2,843 eligible individuals aged 40 years or older. The diagnosis of stage 1 hypertension was systolic blood pressure (SBP) 130~139 mmHg and/or diastolic blood pressure (DBP) 80~89 mmHg. We collect data through standardized questionnaires and clinical and biochemical measurements conducted in unified institutions. The relationship between lipid parameters and risk of stage 1 hypertension were estimated using multiple Cox regression analysis.

Results: During the follow-up period, 388 subjects (13.7%) developed stage 1 hypertension. Incidence of stage 1 hypertension gradually increased with the quartiles of triglycerides (TG) and the ratio of TG to high-density lipoprotein cholesterol (TG/HDL-C, all *P* < 0.0001). Multiple Cox regression analyses indicated that, compared to the quartile 1 of TG/HDL-C, quartile 4 was associated with higher risk of incident stage 1 hypertension (model 1: HR = 2.06, 95% CI 1.49–2.84; model 2: HR = 1.95, 95% CI 1.41–2.70; model 3: HR = 1.48, 95% CI 1.05–2.09).

Conclusions: Our study findings suggest that TG/HDL-C is independently associated with the incidence of stage 1 hypertension in the Chinese middle-aged and elderly population.

## INTRODUCTION

Hypertension is an increasingly serious global health problem and a significant risk factor for heart failure, myocardial infarction, stroke, atrial fibrillation, and chronic kidney disease [1, 2]. According to statistics, as of 2018, the prevalence of hypertension among Chinese adults was 24.7%, with a staggering 274 million hypertensive patients in this age group, of which even 240 million have inadequate control [3]. Hypertension has emerged as a significant public health issue, greatly increasing the healthcare management burden. In the past few years, there has been a significant increase in the Chinese government and public health institutions’ commitment to enhancing hypertension management through the implementation of various policies [4].

In 2017, the American College of Cardiology (ACC) and the American Heart Association (AHA) drew up new hypertension guidelines, lowering the diagnostic threshold and defining the blood pressure range of 130–139/80–89 mmHg as stage 1 hypertension [5]. However, the debate over whether to apply the diagnosis of stage 1 hypertension and lower the blood pressure threshold for diagnosing hypertension has been continuing. One of the main reasons for the above debate is that diagnosing stage 1 hypertension will inevitably increase the prevalence of hypertension and add to the burden of healthcare [6]. Actually, multiple epidemiological evidence has shown that individuals with stage 1 hypertension are at a significantly higher risk of cardiovascular events and all-cause mortality compared with those with normal blood pressure [7–9]. However, other authoritative hypertension guidelines worldwide, such as European Society of Hypertension (ESH), International Society of Hypertension (ISH) and National Institute for Health and Care Excellence (NICE) still recommend the cutoff for hypertension as 140/90 mmHg [10–12]. The difference in hypertension diagnostic criteria between ACC/AHA and other hypertension guidelines precisely lies in the classification of stage 1 hypertension. Currently, the United States has taken the lead in adopting blood pressure higher than 130/80 mmHg as the diagnostic criteria for hypertension [13].

The latest hypertension guidelines in China have not yet included the definition of stage 1 hypertension as a diagnostic criterion for hypertension [14]. However, studies by QI et al. have shown that individuals with stage 1 hypertension in the Chinese population have a markedly higher risk of atherosclerotic cardiovascular disease (ASCVD), coronary heart disease, and stroke [15]. Subsequent studies conducted in various populations and regions have also reinforced the existence of a relationship between stage 1 hypertension and cardiovascular disease (CVD) [16–18]. Therefore, the introduction of stage 1 hypertension diagnosis into China and the update of Chinese hypertension guidelines require more epidemiological evidence from the Chinese population. Currently, there is still a shortage of studies exploring the etiology and risk factors for stage 1 hypertension in China. In accordance with previous definitions, lipid metabolism abnormalities are closely associated with hypertension [19–21]. However, the association between specific lipid parameters and the onset of hypertension is still controversial. Chen et al. observed that higher total cholesterol (TC), low-density lipoprotein cholesterol (LDL-C), and non-high-density lipoprotein cholesterol (non-HDL-C) are linked to a higher risk of hypertension [22]. On the other hand, He et al. found that higher triglycerides (TG) and lower HDL-C are more associated with a higher risk of hypertension [23]. Even some studies have shown that there is no statistically significant association between lipid parameters and stage 1 hypertension [24]. Moreover, lipid parameters in many previous studies did not include lipid ratios. In clinical practice, compared with individual lipid parameters, lipid ratios have stronger predictive power [25, 26]. In summary, the relationship between lipid parameters and stage 1 hypertension remains unclear and requires more evidence from cohort studies.

Dyslipidemia is also an important risk factor for ASCVD, and a better understanding of the impact of dyslipidemia on blood pressure can aid in the overall prevention and treatment of ASCVD [27]. Moreover, investigating the relationship between dyslipidemia and hypertension after relaxing the diagnostic criteria for hypertension helps to understand the role of lipids in the development and progression of hypertension. We believe that different lipid parameters have varying effects on blood pressure. Therefore, the study was conducted in a large community cohort to better assess the impact of dyslipidemia on stage 1 hypertension, we conducted this study.

## METHODS

### Population and study design

**Figure 1:**
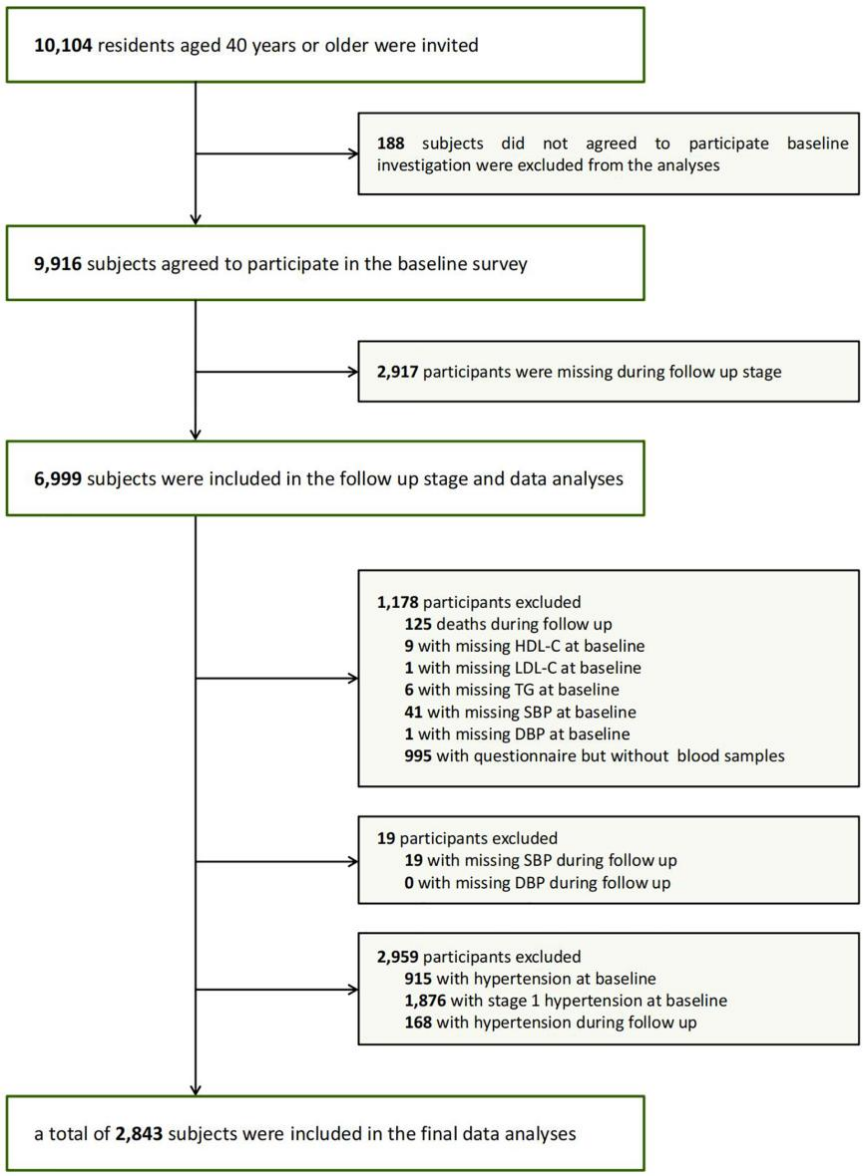
Flowchart of the population selection of the study.

Several communities in Guangzhou, China, participated in this cohort study from 2011 to 2014–2016. The study population was from the Risk Evaluation of cAncers in Chinese diabeTic Individuals: A lONgitudinal (REACTION) study [28, 29]. The recruitment process involved notifying examinations or conducting home visits to recruit 10,104 participants aged 40 years or older (Figure 1). Among these participants, a total of 9,916 participants successfully filled out the informed consent form and were involved in the survey, leading to a participation rate of 98.1%. During the follow-up period from 2011 to 2016, although 2,917 participants were lost to follow-up, 6,999 participants were successfully followed up. 6,999 participants included 125 deaths, 995 participants who were unable to attend in-person to complete a questionnaire survey. Finally, 5,879 participants are currently present in the follow-up database. Therefore, the follow-up rate reached 71%. Among these participants, participants were excluded from the analysis for the following reasons: missing baseline data for HDL-C (*n* = 9), TC (*n* = 0), LDL-C (*n* = 1), TG (*n* = 6), SBP (*n* = 41) or DBP (*n* = 1); missing follow-up data for SBP (*n* = 19) and DBP (*n* = 0); diagnosis of hypertension (*n* = 915) or stage 1 hypertension (*n* = 1876) at baseline; diagnosed of hypertension during follow-up. Participants with missing primary indicators (lipids parameters and blood pressure) were directly excluded from this study, while those with missing non-primary indicators (other indicators) were uniformly included in the analysis. Finally, a total of 2,843 participants satisfied the requirements for the final analysis. The protocol for this study was approved by the Institutional Review Board of Sun Yat-sen Memorial Hospital, Sun Yat-sen University.

### Clinical and biochemical measurements

To collect information on lifestyle, medical history, family history and sociodemographic characteristics for standardized analysis, we used a questionnaire based on specific criteria including the following: gender; age; history of diabetes; history of dyslipidemia; behaviors related to alcohol and tobacco consumption: “never” (no history of smoking or drinking), “current” (regular smoking or drinking within the past 6 months), or “ever” (having quit smoking or drinking for more than 6 months). Additionally, we used a modified version of International Physical Activity Questionnaire (IPAQ) to assess physical activity during leisure time, including questions about the frequency and duration of moderate and vigorous physical activity as well as walking. Separate metabolic equivalent hours per week (MET-h/week) were used to calculate the total amount of physical activity. After the completion of data entry for the questionnaires, methods such as verification and double entry are used to check the accuracy of the data.

In accordance with a standardized protocol, all participants underwent anthropometric examinations under the guidance of well-trained staff. A single observer took three duplicate blood pressure measurements, with a 10-minute gap between each measurement. The blood measurements were all taken with an automatic electronic device (OMRON, Omron Corporation, Shanghai, China). We took the average of blood pressure values measured three times as the final data (SBP. DBP and MAP) included in the statistics. The participants were given instructions to take off their shoes and put on comfortable indoor clothing for measuring height and weight. There was a 0.1 cm and 0.1 kg accuracy in measuring height and weight, respectively. The body mass index (BMI) is calculated by dividing body weight (in kg) by the square of height (in m). Afterwards, the participants stood up and their waist circumference (WC) was measured at the level of the belly button after a gentle exhale.

After participants fasting for at least 10 hours overnight, venous blood samples were collected for laboratory testing. Levels of fasting serum insulin, fasting plasma glucose (FPG), γ-gamma-glutamyl transpeptidase (γ-GGT), TG, TC, HDL-C, LDL-C were measured using an automated analyzer (Beckman CX-7 Biochemical Autoanalyzer, Brea, CA, USA). The non-HDL-C level was calculated by subtracting HDL-C from TC. The non-HDL-C/HDL-C ratio is a measure where non-HDL-C is divided by HDL-C, and the TG/HDL-C ratio is a measure where TG is divided by HDL-C similarly. Hemoglobin A1c value (HbA1c) was measured through high-performance liquid chromatography (Bio-Rad, Hercules, CA, USA).

### Definition of stage 1 hypertension

The diagnosis of stage 1 hypertension was based on the 2017 ACC/AHA guideline: SBP 130~139 mmHg and/or DBP 80~89 mmHg [5].

### Statistical analysis

The analysis of the data was conducted using SAS version 9.4 (SAS Institute Inc., Cary, NC, USA). For variables with bias, the median (quartile range) was used to represent the data. Categorical variants were presented in figures or scales. The mean ± standard deviation (SD) was used to indicate Sequential variants. One-way ANOVA was employed to assess intergroup differences. The χ^2^ test was utilized to compare categorical variables.

A chi-square test was conducted to determine the incidence of stage 1 hypertension in different quartiles of baseline lipid parameters. To assess the relationship between baseline lipid parameters and SBP, DBP as well as mean artery pressure (MAP) at follow-up, Pearson’s correlation and multiple regression models were used. In order to evaluate the association between baseline lipid profiles and the incidence of stage 1 hypertension, multiple Cox regressions were conducted. Based on each of the three regression models, hazard ratios (HRs) and 95% confidence intervals (CIs) were calculated for stage 1 hypertension. The first model was not adjusted, the second model was adjusted for age, and the third model was additionally adjusted for age, sex, BMI, current smoking status, current drinking status, physical activity level, and previous dyslipidemia diagnosis. Because TG is not normally distributed, it is necessary to perform a logarithmic transformation on TG before conducting statistical analysis. The status of tobacco and alcohol consumption (noncurrent/current) were considered as categorical variables.

At follow-up, a subgroup analysis was performed to assess the risk of stage 1 hypertension as the TG/HDL-C interquartile increased across various subgroups. The same factors were taken into account as in Model 3 when adjusting for the model. According to median age (57 years), age was divided into two subgroups. Gender was categorized as male or female. BMI was categorized into normal, overweight, and obese subgroups. In addition, subgroups were divided based on the presence or absence of central obesity, diabetes, and dyslipidemia. In interaction studies, potential factors that could modify the relationship between the incidence of stage 1 hypertension and lipid profiles were investigated separately. To assess interactions, the final model was constructed from the stratum parameters and the interquartile lipid parameters as well as the corresponding interaction terms which involve multiplying the stratification parameter by the lipid parameter interquartile.

All statistical results are two-tailed tests, and a *p*-value less than 0.05 is considered statistically significant.

### Availability of data and materials

The datasets collected and/or analyzed during the current study will be available from the corresponding author upon reasonable request.

## RESULTS

### Characteristics of the participants

Characteristics of the study population at baseline, stratified by stage 1 hypertension status at follow-up, are presented in Table 1. After an average follow-up duration of 3.6 ± 0.7 years, the age of the 2,843 participants was 54.1 ± 6.7 years, and the incidence of stage 1 hypertension was 13.65% (388 participants). Baseline SBP, DBP, MAP, BMI and WC were significantly higher in participants with stage 1 hypertension than those without (all *p* < 0.0001). Baseline biochemical parameters such as TG, non-HDL-C/HDL-C, TG/HDL-C, FPG, HbA1c, fasting insulin, and γ-GGT were also significantly higher in participants with stage 1 hypertension compared to those without (all *p* < 0.0001).

**Table 1:** Characteristics of study population at baseline by stage 1 hypertension status at follow up.

	**Normal reference ranges (Clinical biochemical indicator)**	**Without stage 1 hypertension**	**With stage 1 hypertension**	***P***
*n* (%)^*^	–	2455 (86.3)	388 (13.7)	<0.0001
SBP (mmHg)	–	113.7 ± 8.4	119.5 ± 6.6	<0.0001
DBP (mmHg)	–	68.7 ± 6.1	72.8 ± 5.0	<0.0001
MAP (mmHg)	–	98.7 ± 7.0	104.0 ± 5.3	<0.0001
TG (mg/dl)	27.5–203.7	99.1 (74.3–141.6)	113.3 (83.2–154.4)	0.0010
TC (mg/dl)	112.0–232.0	199.4 ± 47.1	200.8 ± 50.1	0.5809
HDL-C (mg/dl)	31.0–75.8	53.2 ± 14.4	51.1 ± 13.9	0.0080
LDL-C (mg/dl)	50.3–140.0	120.6 ± 36.3	122.2 ± 38.0	0.3997
Non-HDL-C (mg/dl)	76.2–179.0	146.2 ± 41.0	149.7 ± 43.4	0.1204
Non-HDL-C/HDL-C	–	2.89 ± 0.97	3.06 ± 0.94	0.0015
TG/HDL-C	–	1.90 (1.31–2.98)	2.18 (1.50–3.45)	0.0001
Age (years)	–	53.9 ± 6.6	55.6 ± 7.3	<0.0001
Male (*n* (%))	–	539 (22.0)	122 (31.4)	<0.0001
BMI (kg/m^2^)	–	22.8 ± 3.1	23.8 ± 3.0	<0.0001
WC (cm)	–	78.9 ± 8.9	81.7 ± 9.0	<0.0001
Current smoking (*n* (%))	–	214 (8.9)	42 (11.1)	0.1860
Current drinking (*n* (%))	–	69 (2.9)	9 (2.4)	0.5738
Physical activity (MET-h/week)	–	21.0 (10.5–49.0)	24.0 (10.5–45.0)	0.9388
FPG (mmol/L)	3.90–5.60	5.28 (4.91–5.69)	5.42 (5.06–5.90)	<0.0001
HbA1c	4.00–6.00	5.80 (5.60–6.10)	5.90 (5.60–6.20)	<0.0001
Fasting insulin (μIU/ml)	3.00–25.00	6.30 (4.80–8.50)	7.20 (5.30–9.70)	<0.0001
γ-GGT (U/L)	10.0–60.0	18.0 (13.0–25.0)	20.0 (15.0–28.0)	<0.0001

Data were means ± SD or medians (interquartile ranges) for skewed variables or numbers (proportions) for categorical variables. ^*^n (%) was for the number of incident stage 1 hypertension status at follow up. *P*-values were for the ANOVA or χ^2^ analyses across the groups. Abbreviations: SBP: systolic blood pressure; DBP: diastolic blood pressure; MAP: mean arterial pressure; TG: triglycerides; TC: total cholesterol; HDL-C: high-density lipoprotein cholesterol; LDL-C: low-density lipoprotein cholesterol; BMI: body mass index; WC: waist circumference; MET-h/week: separate metabolic equivalent hours per week; FPG: fasting plasma glucose; γ-GGT: γ-glutamyl transferase.

### Relationship between baseline lipid parameters and blood pressure at follow-up

Table 2 presents the correlations between blood pressure (SBP, DBP, and MAP) and different baseline lipid metabolism parameters (TG, TC, HDL-C, LDL-C, non-HDL-C, non-HDL-C/HDL-C, TG/HDL-C) at the follow-up stage. There were significant positive correlations of blood pressure (SBP, DBP, and MAP) with TG, non-HDL-C/HDL-C and TG/HDL-C (all *p* < 0.0001). Conversely, there were significant negative correlations of blood pressure (SBP, DBP, and MAP) with HDL-C (all *p* < 0.001). In the multivariate regression analysis and after adjusting for age and gender as confounding variables, baseline TG, HDL-C, non-HDL-C/HDL-C, and TG/HDL-C stayed robust and independent determinants of follow-up stage SBP, DBP, and MAP (all *p* < 0.0001).

**Table 2:** Pearson’s correlation and multiple regression analysis of baseline lipid parameters associated with SBP, DBP and MAP at follow up.

	**SBP (mmHg)**				**DBP (mmHg)**				**MAP (mmHg)**			
	**r**	***P***	**St. β**	***P***	**r**	***P***	**St. β**	***P***	**r**	***P***	**St. β**	***P***
TG (mg/dl)	0.14	<0.0001	0.12	<0.0001	0.12	<0.0001	0.12	<0.0001	0.15	<0.0001	0.13	<0.0001
TC (mg/dl)	0.03	0.1621	0.01	0.5290	−0.01	0.7599	0.01	0.4306	0.02	0.3060	0.01	0.4745
HDL-C (mg/dl)	−0.10	<0.0001	−0.09	<0.0001	-0.11	<0.0001	−0.09	<0.0001	−0.11	<0.0001	−0.09	<0.0001
LDL-C (mg/dl)	0.05	0.0045	0.04	0.0540	0.02	0.3428	0.03	0.0788	0.05	0.0120	0.04	0.0447
Non-HDL-C (mg/dl)	0.07	0.0002	0.05	0.0133	0.04	0.0538	0.05	0.0072	0.06	0.0006	0.05	0.0070
Non-HDL-C/HDL-C	0.16	<0.0001	0.12	<0.0001	0.14	<0.0001	0.13	<0.0001	0.16	<0.0001	0.13	<0.0001
TG/HDL-C	0.16	<0.0001	0.14	<0.0001	0.15	<0.0001	0.14	<0.0001	0.17	<0.0001	0.15	<0.0001

Abbreviations: SBP: systolic blood pressure; DBP: diastolic blood pressure; MAP: mean arterial pressure; TG: triglycerides; TC: total cholesterol; HDL-C: high-density lipoprotein cholesterol; LDL-C: low-density lipoprotein cholesterol; r: correlation coefficient; St. β: Standardized regression coefficient. All parameters were logarithmically transformed prior to analysis due to non-normal distributions. Multiple regression analysis is adjusted for age and sex.

### Relationship between baseline lipid parameters and risk of stage 1 hypertension

**Figure 2:**
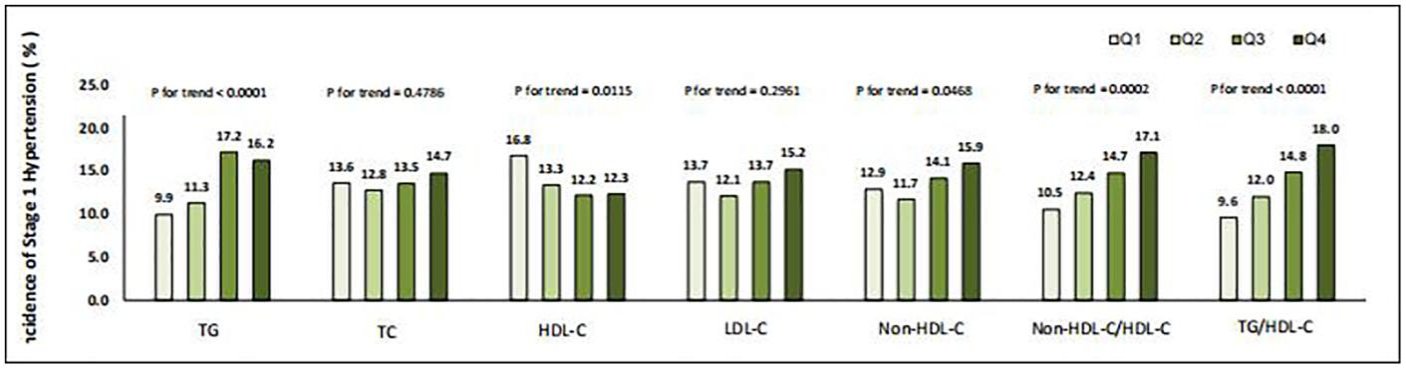
Incidence of stage 1 hypertension in different quartiles of baseline lipid parameters.

In Figure 2, the incidence of stage 1 hypertension rises with the increase in quartiles of TG, non-HDL-C, non-HDL-C/HDL-C, and TG/HDL-C, and decreases with the increasing HDL-C quartiles (all *p* < 0.05). Among them, the most significant trend is the incidence of stage 1 hypertension with the increase in quartiles of TG/HDL-C (*p* < 0.0001).

Table 3 presents the HRs and 95% CIs of stage 1 hypertension with different quartiles of TG, TC, HDL-C, LDL-C, non-HDL-C, non-HDL-C/HDL-C and TG/HDL-C in the overall study population across three different models. The stability of the findings was confirmed through multivariable adjusted Cox regression analysis. TG/HDL-C presented the most significant correlation with stage 1 hypertension among all lipid indicators in all Cox regression models. In model 3, after adjusting for age, sex, BMI, current smoking status, current drinking status, physical activity level, and previously diagnosed dyslipidemia, the HRs for stage 1 hypertension associated with increasing quartiles of TG/HDL-C were 1 (reference), 1.10 (95% CI 0.77–1.57), 1.32 (95% CI 0.94–1.86) and 1.48 (95% CI 1.05–2.09).

**Table 3:** Association between baseline lipid parameters and risk of incident stage 1 hypertension.

		**Quartile 1**	**Quartile 2**	**Quartile 3**	**Quartile 4**	**1-Quartile change^#^**
TG	Model 1	1	1.15 (0.82–1.62)	1.88 (1.37–2.58)	1.75 (1.28–2.41)	1.24 (1.12–1.36)
	Model 2	1	1.12 (0.80–1.57)	1.76 (1.28–2.43)	1.64 (1.19–2.26)	1.21 (1.09–1.33)
	Model 3	1	1.14 (0.81–1.63)	1.72 (1.24–2.40)	1.40 (1.00–1.96)	1.14 (1.03–1.27)
TC	Model 1	1	0.93 (0.68–1.26)	0.99 (0.73–1.35)	1.10 (0.81–1.48)	1.04 (0.94–1.14)
	Model 2	1	0.90 (0.66–1.23)	0.95 (0.70–1.28)	1.00 (0.74–1.36)	1.01 (0.91–1.11)
	Model 3	1	0.88 (0.64–1.21)	1.02 (0.75–1.40)	1.09 (0.80–1.49)	1.04 (0.94–1.15)
HDL-C	Model 1	1	1.31 (0.97–1.76)	1.45 (1.08–1.95)	1.43 (1.06–1.93)	1.13 (1.03–1.25)
	Model 2	1	1.34 (0.99–1.80)	1.48 (1.10–1.99)	1.44 (1.07–1.95)	1.14 (1.03–1.25)
	Model 3	1	1.20 (0.88–1.63)	1.29 (0.95–1.78)	1.04 (0.75–1.45)	1.03 (0.93–1.14)
LDL-C	Model 1	1	0.86 (0.63–1.18)	1.00 (0.74–1.35)	1.13 (0.84–1.51)	1.05 (0.96–1.16)
	Model 2	1	0.84 (0.61–1.14)	0.94 (0.69–1.28)	1.02 (0.76–1.38)	1.02 (0.93–1.12)
	Model 3	1	0.85 (0.61–1.18)	1.02 (0.74–1.39)	1.06 (0.77–1.45)	1.04 (0.94–1.15)
Non-HDL-C	Model 1	1	0.90 (0.66–1.24)	1.12 (0.82–1.51)	1.28 (0.95–1.72)	1.10 (1.00–1.21)
	Model 2	1	0.87 (0.63–1.19)	1.04 (0.77–1.42)	1.17 (0.87–1.58)	1.07 (0.97–1.18)
	Model 3	1	0.87 (0.62–1.21)	1.07 (0.78–1.47)	1.18 (0.86–1.61)	1.08 (0.97–1.19)
Non-HDL-C/HDL-C	Model 1	1	1.21 (0.87–1.67)	1.47 (1.07–2.02)	1.75 (1.28–2.40)	1.21 (1.10–1.33)
	Model 2	1	1.18 (0.85–1.63)	1.39 (1.01–1.91)	1.63 (1.19–2.23)	1.18 (1.07–1.30)
	Model 3	1	1.08 (0.77–1.52)	1.15 (0.83–1.62)	1.28 (0.91–1.79)	1.08 (0.97–1.21)
TG/HDL-C	Model 1	1	1.28 (0.91–1.81)	1.64 (1.18–2.27)	2.06 (1.49–2.84)	1.27 (1.15–1.41)
	Model 2	1	1.23 (0.87–1.74)	1.56 (1.12–2.16)	1.95 (1.41–2.70)	1.25 (1.13–1.38)
	Model 3	1	1.10 (0.77–1.57)	1.32 (0.94–1.86)	1.48 (1.05–2.09)	1.15 (1.03–1.28)

Data are hazard ratios (95% confidence interval). Participants without stage 1 hypertension at follow up are defined as 0 and with stage 1 hypertension as 1. ^#^All variables were calculated for 1-Quartile increasing of lipid parameters except for HDL-C, which was calculated for 1-Quartile decreasing. Model 1 is unadjusted. Model 2 is adjusted for age. Model 3 is adjusted for age, sex, BMI, current smoking status, current drinking status, physical activity level and previously diagnosed dyslipidemia.

**Figure 3:**
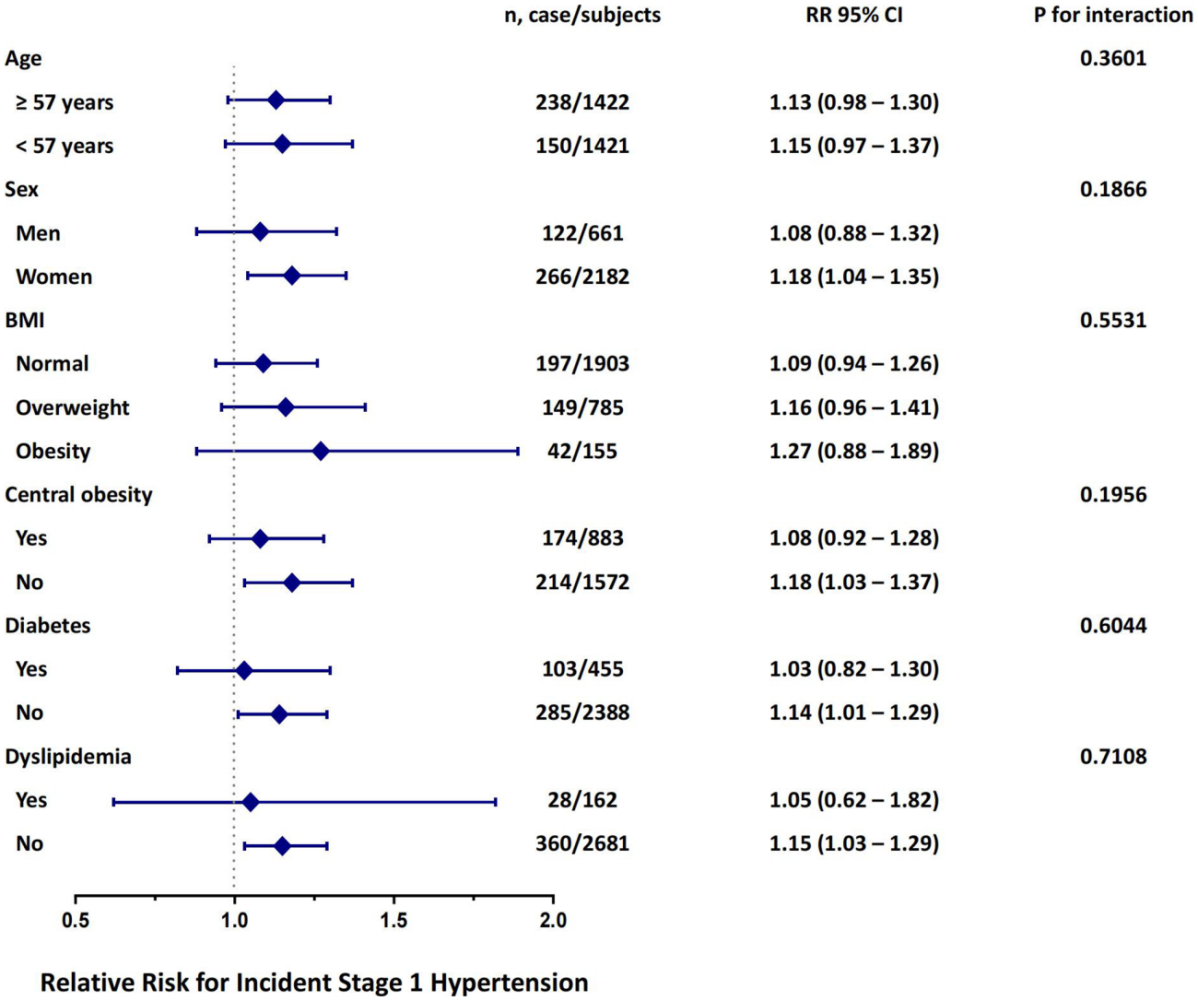
**Risk of incident stage 1 hypertension with each quartile increase of TG/HDL-C in different subgroups at follow up.** The model is adjusted for age, sex, BMI, current smoking status, current drinking status, physical activity level and previously diagnosed dyslipidemia.

Multivariate-adjusted HRs of increased TG/HDL-C quartiles with incident stage 1 hypertension in different subgroups are presented in Figure 3. In subgroup analyses, a statistically marked correlation between TG/HDL-C and the risk of stage 1 hypertension was found in subgroups of females, individuals without central obesity, non-diabetic individuals and those without dyslipidemia. Notably, the interaction analyses of all subgroups were not statistically significant (all *p* for interaction >0.05), which indicated that the relationship between TG/HDL-C and the risk of stage 1 hypertension is not meaningfully influenced by age, sex, BMI, central obesity, diabetes, or dyslipidemia status in this study population.

## DISCUSSION

The current research findings suggest that TG/HDL-C is a more effective predictor of stage 1 hypertension than other commonly used lipid indicators. This is the first large-scale prospective study to demonstrate that TG/HDL-C has a predictive value of incident stage 1 hypertension among those with normal blood pressure. These results emphasize the significance of paying close attention to TG/HDL-C in clinical practice to reduce the risk of developing stage 1 hypertension.

Our study has confirmed the correlation between dyslipidemia and the incidence of stage 1 hypertension in the Chinese population. Currently, there are no globally unified diagnostic criteria for hypertension, and the primary disagreement revolves around the classification of stage 1 hypertension as hypertension or not. Dyslipidemia and blood pressure abnormalities are the two key factors leading to ASCVD, and they synergistically increase the risk of cardiovascular events and mortality [30]. Our study further confirms the above viewpoint in the Chinese population, that there is a clear correlation between dyslipidemia and early mild elevation of blood pressure. This allows us to better understand the contribution of lipid metabolism disorders to early blood pressure elevation. Thus, it is indeed necessary to reconsider whether the current diagnostic criteria for hypertension should be lowered to include stage 1 hypertension.

In this study, we observed that higher TG levels and lower HDL-C concentrations were correlated with a higher risk of stage 1 hypertension. However, the correlation between TG and HDL-C with the incidence of stage 1 hypertension was not as significant as the correlation between TG/HDL-C and the risk of stage 1 hypertension, demonstrating that TG/HDL-C as an independent indicator has better predictive value for incident stage 1 hypertension. Previous studies examining the correlation between lipid metabolism and the risk of hypertension did not consider ratios such as non-HDL-C/HDL-C and TG/HDL-C [31, 32]. Our previous research has also demonstrated the independent association of TG/HDL-C ratio with the incidence of chronic kidney disease (CKD) in the Chinese adults, rather than other lipid parameters [33]. This suggests that lipid metabolism parameters themselves may have strong predictive capabilities, but appropriate data processing is required. Further research is necessary to explore the potential clinical significance of lipid metabolism indicator ratios, such as TG/HDL-C and non-HDL-C/HDL-C, which may be more important than commonly used single lipid indicators. Previous studies have demonstrated that the TG/HDL ratio is a robust predictor of insulin resistance [34, 35]. Che et al. have also confirmed that the TG/HDL ratio, as a conveniently measurable alternative indicator, can predict the occurrence of CVD. They posit that the impact of the TG/HDL ratio on CVD is closely associated with hypertension and dyslipidemia [36]. These findings collectively substantiate the predictive value of the TG/HDL ratio in metabolic diseases and CVD, thereby enhancing the credibility of our research findings.

However, different populations and patterns may show inconsistent relationships between lipid metabolism indicators and hypertension. Several cross-sectional studies in China have shown that the correlation between HDL-C and the incidence of hypertension changes from negative to positive after HDL-C is adjusted for BMI [31, 37, 38]. This is inconsistent with the results of other studies, where HDL-C is negatively correlated with the risk of stage 1 hypertension, and HDL-C is also negatively correlated with SBP, DBP, and MAP. It may be due to the fact that these studies are all cross-sectional studies, which cannot clearly analyze the correlation between lipid metabolism indicators and the risk of hypertension. It deserves to be taken into account that the observed outcome of this study being stage 1 hypertension, which represents a lower blood pressure threshold for hypertension, may have influenced the reactivity and sensitivity of lipid metabolism indicators. Different blood pressure levels could potentially impact the relationship between these indicators and hypertension.

The correlation between lipid indicators and the onset of stage 1 hypertension is complex while dyslipidemia can contribute to the occurrence and development of stage 1 hypertension through multiple mechanisms. Hypertriglyceridemia can lead to endothelial dysfunction, and decreased HDL-C can reduce the bioavailability of nitric oxide (NO) in endothelial cells [39], thereby impairing vasodilation and causing an increase in blood pressure. Dyslipidemia also contributes to higher level of endothelin-1 in circulation [40], which has mitogenic and vasoconstrictive properties, promoting the development of hypertension [41]. Hypertriglyceridemia can lead to the accumulation of lipid metabolites in hepatocytes, resulting in hepatic insulin resistance. Insulin resistance can reduce the production of NO [42] and also over activate the sympathetic nervous system and the renin-angiotensin system [43], all of which contribute to vasoconstriction and elevated blood pressure.

This study has certain limitations. Firstly, we only measured lipid levels and blood pressure at one time point, which may not accurately reflect the fluctuations that can occur in lipid levels and blood pressure in real-life situations. However, our blood pressure results are based on the average of three measurements, and the blood lipid measurements are conducted in a central laboratory, ensuring stable and reliable results. This method has also been chosen in previous large-scale epidemiological studies. Secondly, other less common lipid parameters, such as lipoprotein(a) and apolipoproteins, were not included in this study’s analysis. Subsequent studies should consider incorporating these lipid parameters. Additionally, the study did not gather data on the usage of lipid-lowering medications or other factors that could impact blood lipid levels in individuals with dyslipidemia. This omission could potentially influence the interpretation of certain findings, but we corrected for past medical history related to dyslipidemia, which might dilute this effect. Furthermore, this study only included the Chinese population, and the study results may not apply to other ethnic groups. The population data were derived from a cohort specifically designed to investigate diabetes and its complications. Hence, future studies should consider selecting participants from the general population to minimize potential selection bias.

## CONCLUSIONS

We have reported a more marked correlation between TG/HDL-C and the incidence of stage 1 hypertension compared with other lipid indicators shown in this study. Our study results indicate that TG/HDL-C may have better predictive value for the incidence of stage 1 hypertension. It may indicate that we should control dyslipidemia at early stage, which could help prevent stage 1 hypertension and reduce the incidence of ASCVD events.
